# A Text Mining Pipeline Using Active and Deep Learning Aimed at Curating Information in Computational Neuroscience

**DOI:** 10.1007/s12021-018-9404-y

**Published:** 2018-11-15

**Authors:** Matthew Shardlow, Meizhi Ju, Maolin Li, Christian O’Reilly, Elisabetta Iavarone, John McNaught, Sophia Ananiadou

**Affiliations:** 1 The National Centre for Text Mining, School of Computer Science, University of Manchester, Road, M13 9PL, Oxford, UK; 2 Blue Brain Project, EPFL, Campus Biotech, Ch. des Mines 9, CH-1202 Geneva, Switzerland

**Keywords:** Text mining, Data mining, Named entity recognition, Corpus, Conditional random field, Deep learning, Annotation, Data curation

## Abstract

The curation of neuroscience entities is crucial to ongoing efforts in neuroinformatics and computational neuroscience, such as those being deployed in the context of continuing large-scale brain modelling projects. However, manually sifting through thousands of articles for new information about modelled entities is a painstaking and low-reward task. Text mining can be used to help a curator extract relevant information from this literature in a systematic way. We propose the application of text mining methods for the neuroscience literature. Specifically, two computational neuroscientists annotated a corpus of entities pertinent to neuroscience using active learning techniques to enable swift, targeted annotation. We then trained machine learning models to recognise the entities that have been identified. The entities covered are Neuron Types, Brain Regions, Experimental Values, Units, Ion Currents, Channels, and Conductances and Model organisms. We tested a traditional rule-based approach, a conditional random field and a model using deep learning named entity recognition, finding that the deep learning model was superior. Our final results show that we can detect a range of named entities of interest to the neuroscientist with a macro average precision, recall and F1 score of 0.866, 0.817 and 0.837 respectively. The contributions of this work are as follows: 1) We provide a set of Named Entity Recognition (NER) tools that are capable of detecting neuroscience entities with performance above or similar to prior work. 2) We propose a methodology for training NER tools for neuroscience that requires very little training data to get strong performance. This can be adapted for any sub-domain within neuroscience. 3) We provide a small corpus with annotations for multiple entity types, as well as annotation guidelines to help others reproduce our experiments.

**Electronic supplementary material:**

The online version of this article (10.1007/s12021-018-9404-y) contains supplementary material, which is available to authorized users.

## Background

Large local and international projects such as the Swiss Blue Brain Project, the European Human Brain Project, the Allen Brain Observatory, and the American BRAIN initiative have recently emerged in neuroscience and are pushing traditional neuroscience toward the big science paradigm (Underwood 2016). At their heart, these projects harness big data to model in great detail the functioning of the brain, down to the level of the individual neuron types. The data-driven approach adopted for such large-scale modelling requires characterization of numerous entities, such as neuron types, synapses, and ion channels. Although the structural and functional aspects of these entities can partly be evaluated in the laboratory, the complexity and the cross-scale nature of the modelled phenomena prohibit a comprehensive evaluation of all aspects at play. Thus, experimental data must be complemented with the scientific knowledge accumulated world-wide and recorded in the scholarly literature. This situation is making increasingly clear the need for large and high-quality databases of literature-curated information about entities related to neuroscience. Crucially, we need to reliably catalogue previously reported experimental measurements so that they can be integrated as values for modelling parameters or be used to validate emergent properties of models. Although recently reaching more awareness, similar needs have long been recognized and previously motivated large and enduring curation projects in the life sciences, such as UniProt for proteins (The UniProt Consortium 2017). For neuroscience, many efforts have been deployed to categorise and define the types of entities described across the neuroscience literature (Larson and Martone 2013). Further, some high-quality databases are now available, such as NeuroElectro (Tripathy et al. 2014), which records a large number of values for a representative, although limited, number of neuronal modelling parameters.

To promote traceability and reusability of systematically curated literature for the large number of parameters needed for detailed data-driven modelling of the brain, a new manual curation framework has been recently proposed (O’Reilly et al. 2017). However, this manual process requires a team of curators to sift through abstracts and full texts to be able to identify new entities and their properties. This is a slow and painstaking process, which requires a curator to maintain a mixture of specialist domain knowledge alongside the necessary informatics knowledge to ensure that they are able to discover all the relevant documents for a new entity. Furthermore, a curator’s work is never finished. The meaning of terms may shift over time and new terms may be created as the collective understanding of the field changes and new material is published. What then can we do to help the beleaguered curator? Text mining can come to their aid. We can use a variety of techniques to lift the burden of informatics from the curator and allow them to focus on applying their own specialist domain knowledge to the entities in question. In this paper, we propose such a system for the curation of several neuroscience entities. By identifying these entities automatically in text, we can help the curator to quickly survey the literature for papers of interest. Further, when processing a paper to extract relevant experimental values, the curator can benefit from these named entity annotations to speed-up the work needed to identify and characterize the context surrounding such experimental values (e.g., cell type, species, brain regions, etc.).

Whereas other research teams have previously reported text mining techniques for neuroscience, such as WhiteText (French and Pavlidis. 2012) or Textpresso (Müller et al. 2008), our methods apply cutting-edge text mining techniques to the neuroscience literature, targeting a wide set of entity types with state of the art performance (as measured by the standard metrics of precision, recall and F-measure). Early work on text mining for neuroscience focussed on the area of document classification (Crasto et al. 2003; Van Driel et al. 2006). In this task, properties of documents and entities are assembled and then processed to provide some meaningful information, such as identifying relations between phenotypes (Van Driel et al. 2006), or to populate neuroscience databases (Crasto et al. 2003). This work was recently further developed and applied to literature surveys (Balan et al. 2014) and information retrieval (Lapish et al. 2013). Document classification is useful when a user needs to find relevant documents or entities from a large collection. However, it can only tell a user which documents contain relevant information, not where such information is localized within the documents.

Named entity recognition can be used to further target the valuable information held in documents. In this technique, a researcher first typically establishes the categories of named entities that they are interested in. They then manually annotate a corpus of documents with examples of the entity types. A NER tool can then be trained using these examples via one of the standard libraries, such as CRF++,^1^ or NERSuite.^2^ The NER tool can then be applied to further documents, or document collections, as a building block for more complex tasks such as curation (Ambert et al. 2013), information retrieval (Müller et al. 2008) and inference (Pan et al. 2006). Notable efforts to create named entity recognition tools for neuroscience have focussed on the identification of brain regions (French et al. 2009; French et al. 2012), neuron types (Ambert et al. 2013), brain connectivity (Vasques et al. 2015; Richardet et al. 2015a), and a number of entities related to spinal cord injuries (Stöckel et al. 2015).

In addition to identifying named entities in a text, researchers may also wish to identify relations between the entities that they extract, adding an extra dimension of information that can help to understand how the entities are being used and, eventually, filter out unwanted entities. In neuroscience, French et al. (2012) used relation extraction techniques to identify connectivity statements between brain regions. This work was continued by Richardet et al. (2013), with some improvements.

Richardet et al. provided the NeuroNER resource (Richardet et al. 2015b), which created a set of rules built on top of UIMA RUTA.^3^ This resource can be applied to a large text corpus to determine the names of neuronal cells within the text. It achieves this through a series of hand crafted rules developed through the collaborative efforts of text mining experts and neuroscientists.

The Textpresso text mining framework for neuroscience (Müller et al. 2008) is designed to help search through neuroscience research papers by providing a semantic search interface. Users can enter names of entities from predefined categories and are shown documents which contain their entity of choice. The system also handles relations, allowing a user to filter documents based on entities occurring in a number of relation types. Evaluation by Balan et al. (2014) showed that Textpresso was useful in the context of performing a literature survey. The Textpresso system relies on a large dictionary of terms, which are matched against the text of documents to identify named entities. This technique is less powerful than the conditional random field (CRF)/deep learning approaches that we have investigated, as it is constrained to identifying entities that occur in the dictionary and cannot identify new occurrences, or variants of terms.

Named entity recognition requires annotated data, which can be time consuming to produce. However, techniques exist to reduce the manual effort otherwise needed to annotate numbers of documents, whilst retaining strong performance. Active learning is a popular method of selecting sentences which will provide the most informative examples for a named entity recognition tool to learn from (Kim et al. 2006; Settles and Craven 2008; Y. Chen et al. 2015; D. Shen et al. 2004), and has previously been applied for the recognition of neuron types (Ambert et al. 2013).

To automatically recognise named entities in text, the standard approach has been to use the CRF (Lafferty et al. 2001). Typical features have included, for each token: the part-of-speech, lemma and other surface features. Standard libraries such as NERSuite and CRF++ are freely available, lowering the barrier to entry for this approach. A new trend in named entity recognition is to use recent developments in the field of deep learning to create contextual embedding representations of a sentence that can be used as input to a CRF. This has been shown to be a very powerful approach, and led to an improved performance on a number of natural language processing tasks (Huang et al. 2015; Rao et al. 2016; D. Chen and Manning 2014), especially when combined with active learning (Y. Shen et al. 2017). Our approach, based upon the work of Lample et al. (2016), is described in further detail in the next section.

Two prominent deep learning algorithms are Long Short Term Memory (LSTM) (Hochreiter and Schmidhuber 1997) and Convolutional Neural Networks (CNN) (Lecun et al. 1998). These vary in implementation and a technical description of their inner workings is outside the scope of this paper. LSTM operates on sequences of data, processing one input at a time and updating a final output state vector depending on the value of the next input. It performs a series of operations that allow it to select the most relevant parts of an input and apply only these in updating its final output. In contrast, CNNs are used to generate multiple sets of features from a particular neighbourhood region of an input, which can be pooled to build strong classifiers. The principal difference between a CNN and a LSTM is that the CNN takes features from a fixed neighbourhood around a target item, whereas the LSTM takes features from an entire sequence. The LSTM is able to use more of the context surrounding a token. For this reason, we have chosen to use LSTMs in our work.

In their seminal work on deep learning for NER, (Lample et al. 2016) presented a novel architecture applying recent advances in the field of deep learning for named entity recognition. Whereas typical approaches for named entity recognition are resource heavy, i.e., requiring lots of domain knowledge to be fed to the classifier, Lample et al. were able to leverage deep learning to create a resource light architecture that gained state of the art performance across several languages. We have adopted the bidirectional LSTM approach put forward by Lample et al. in our work. Our approach is described in full in the methods section of this paper.

Recently, several researchers have applied advances in the field of deep learning to various problems within neuroscience. Advances in image classification have been applied for neuroimaging (Plis et al. 2014), with applications to functional and structural brain imaging data. Later work has attempted to use advancements in the field of deep learning to model and understand neural responses (Yamins and DiCarlo 2016). Finally, neural networks have been used to understand the brain as a computational model (Marblestone et al. 2016), where the authors present a series of hypotheses about how a brain can be understood using the principles of optimisation found in neural networks.

## Methods

### Entities of Interest and Annotation Rules

For this work, we annotated only entities that are specific members of a generic class (e.g., ‘pyramidal neurons’ are a specific member of the generic class ‘neuron type’). Mentions of the generic class (e.g., “These *neurons* are known to be […]”) have not been annotated, only specific entities (e.g., “The input resistance of *thalamo-cortical cells* […]”). Adjective forms (e.g., cortical) of corresponding entities (e.g., cortex) have not been separately annotated. In many instances, boundaries defining an entity were found to be intrinsically open to interpretation by different annotators Thus, to provide reproducible manual annotations, we found it necessary to define clear annotation rules. Although some of these rules may appear subjective or arbitrary, they were necessary to clarify borderline cases. These rules were established empirically by common agreement between the annotators (COR, EI) and text-miners (Limsopatham and Collier. 2016). Below, we summarize the most important aspects of these guiding annotation rules for the six types of entities that we targeted. The complete annotation guidelines are available as part of the supplementary material to this work.

#### Neuron Types

Mention of types of neurons (e.g., *Martinotti cells*), the electrically excitable cells of the nervous system. We included in the annotation all the qualifiers that define the cell types, for example the brain region (e.g., *thalamo-cortical relay cell of the ventrobasal complex*).

#### Brain Regions

We annotated any sequence of tokens referring to a specific area of the brain. This includes for example cortical layers (e.g., *layer 1 of the cortex*), areas mentioned by their function (e.g., *somatosensory area of the thalamus*), but excludes the mention of a system (e.g., the somatosensory system) or of a “representation” (e.g., the shoulder representation).

#### Experimental Values

This is a broad class of quantifiable values (including the unit, if present) that can be reported in a paper, defining either the experimental context or the results of an experiment. It could be defining a range (e.g., *−100 to − 40 mV*) or be a list of values all related to the same entities such as repeated measurements. However, two values separated by a conjunction (e.g. “with *0.13* and *0.20 ms* respectively”) have been annotated separately if referring to different entities (e.g., two different cell types). Sample sizes were not annotated as an experimental value. We do not record the underlying variables that the experimental value refers to. This is left for future work.

#### Units

This type relates to units which describe a scientific quantity, possibly as defined by the International System of Units. These often occur as abbreviations. A unit can in some cases be a noun or phrase that is not typically thought of as a unit (e.g., 20 *spikes per stimulation period*).

#### Ion Currents, Channels, and Conductances

These three entities have been distinguished and annotated separately to help better define their scope. They are closely related entities (i.e., an ion current is generated by the flow of ions through ion channels, and the behaviour of the channel can be modelled by its conductance), thus often the annotations of these three entities are very similar, just differing by their last part (e.g., *T-type Ca2+ channels, T-type Ca2+ currents, T-type Ca2+ conductance*). These entities can often be referred to by the name of their protein (e.g., *Cav3.1 ion channels*) or of their coding gene (e.g., *CACNA1G*). References to only a sub-domain of an ion channel have not been annotated, unless they were used to refer to a channel variety (e.g., *alpha1G*, a type of alpha subunit, is often used as synonym for *Cav3.1 ion channels*). In general, alpha sub-units can give their name to the channel type, not beta sub-units, at least for sodium and calcium channels. In case of genetically modified animals referred to as gene_name−/− where the gene_name is associated with a knock-down ion channel, we annotated the gene_name as a reference to an ion channel (e.g., *“Both tonic and burst spikes were observed in CaV3.1+/+ TC cells, whereas only tonic spikes were detected in CaV3.1−/− TC cells”*).

#### Model Organism

We use the entity model organism as a broad concept, not distinguishing species, strains, or genetic mutation. Similarly, we also use this entity to annotate classes of species (e.g., *rodent*). Model organisms may be referred to via an informal name (e.g., *rabbit*) as well as the formal Latin name (e.g., *Oryctolagus cuniculus*). Strings with implicit mention of a model organism have also been annotated. This is the case for example when talking about a strain and its wild-type counterpart: wild-type is not in itself a species but is referring to a species entity (i.e., the wild-type counterpart of an experimental strain previously mentioned). For the same reason, “*normal rat*” was annotated (including the “*normal*” qualifier) when used to contrast to another strain (e.g., *Genetic Absence Epilepsy Rat from Strasbourg*) and is implicitly used as a synonym for “*wild type*”.

### Comparison to Textpresso

Another system which identifies a range of neuroscience categories is the Textpresso for Neuroscience system. We have described this system in the background section. Here, we will provide a comparison of the categories offered by Textpresso, compared to those that we have proposed. The nine categories offered by Textpresso for Neuroscience are: ‘Brain Area’, ‘Drugs of Abuse’, ‘Nicotine Addiction (NICSNP) Candidate Gene’, ‘NIF Cell Type’, ‘Neuropsychology and Behaviour’, ‘Prescription Drug of Abuse’, ‘Receptor’, ‘Substance Abuse’ and ‘TRP Channel’. Our categories differ in that they are much more general than these.

### Corpus Annotation

To ensure that the solution we developed is useful and applicable for real-life projects, all designing steps of this project have been informed by an existing use-case, the ongoing effort at the Blue Brain Project to develop a biophysically-detailed model of the rodent thalamocortical loop. Hence, the papers used for building our corpus of annotations were drawn from a set of papers selected by a computational neuroscientist (COR) as those that have been previously the most manually annotated with NeuroCurator. The selected papers have been manually selected because they contain useful experimental values for this specific use-case. Thus, this is an application-centric corpus, not aimed at providing exhaustive or unbiased representation across brain regions, cell types, or species. Such generality is not needed here since the goal pursued by this project is to develop a NER Tool designing approach that can be re-used for specific applications, using small size corpora of manual annotations and active learning.

To develop this corpus, we initially ran a pilot annotation with one annotator (COR) of 15 abstracts from neuroscience articles drawn from the set previously described. We used this pilot study to understand the requirements of the annotations for the targeted use case and to give the curator some training material so that they could familiarize themselves with the annotation tool. This preliminary phase was also essential to create clear annotation guidelines that define the scope of the entities, thereby promoting high inter-annotator agreement. We used the brat rapid annotation tool (Stenetorp et al. 2012) for all our annotations. For our next round of annotation, we used active learning. We collected 160 abstracts from our initial set and processed these using the active learning selection criteria described below. We were able to use the 15 abstracts we had annotated earlier as the seed documents for our active learning method. The top 500 sentences were selected after active learning and were annotated by our annotator. In our final round of annotation, we collected 15 full text papers related to neuroscience. We used the 500 sentences that had been annotated in the first round as our seed set for the second round of active learning. The DOI (or PubMed ID when DOI were not available) for the 15 first abstracts and for the 15 full texts are provided in Table 1.

**Table 1:** DOI/PMID for the first set of 15 abstracts and the 15 full text papers

Abstracts	Full text
10.1523/JNEUROSCI.2740-15.2015	10.1002/cne.22461
10.1093/cercor/bhr356	10.1073/pnas.1320572110
10.1152/jn.00647.2013	10.1093/cercor/bhr356
PMID_7965855	10.1113/jphysiol.2004.070888
PMID_9570789	10.1152/jn.00647.2013
10.1152/physrev.00012.2003	10.1152/jn.00926.2014
10.1073/pnas.1320572110	10.1152/physrev.00012.2003
10.1002/cne.22461	10.1523/JNEUROSCI.2740-15.2015
10.1113/jphysiol.2004.070888	10.1523/JNEUROSCI.17-09-03215.1997
10.1007/s12264-013-1402-3	10.1371/journal.pone.0107780
PMID_9518268	10.1007/s00424-012-1188-6
PMID_9457638	10.1523/JNEUROSCI.2333-06.2006
PMID_10408596	10.1113/jphysiol.2002.030643
10.1152/jn.00926.2014	10.1523/JNEUROSCI.3073-10.2011
PMID_9096155	10.1523/JNEUROSCI.0607-16.2016

To ensure consistency in our annotations we asked a second annotator (EI, also a computational neuroscientist) to doubly annotate the first 200 sentences from each of the two active learning rounds. This allowed us to calculate inter-annotator agreement using F1-measure. Through discussions of discrepancies between the two sets of annotations, we noted a number of areas for clarification, which were incorporated in the guidelines. All other annotations were updated as the guidelines were updated. Aside from consulting on borderline or ambiguous cases, both curators performed their work blindly with respect to (1) each other’s annotations, (2) the results of inter-annotator agreement and (3) the results of named entity recognition tools that were created using the corpus that was being produced.

**Fig. 1:**
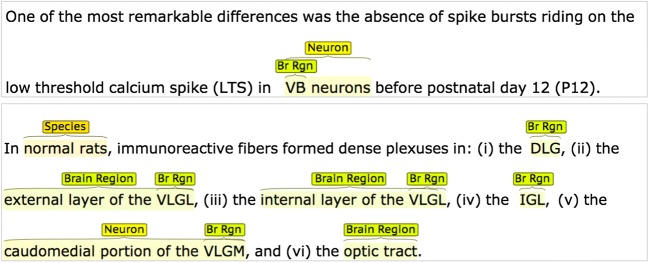
2 example sentences with annotations as they appeared in the brat rapid annotation tool

Figure 1 shows 2 examples of manually created annotations within the brat tool. The first example shows annotations that cover a neuron type and a brain region. We chose to annotate ‘VB’ as a brain region when it occurred describing the location of neuron types and applied this rule consistently throughout our corpus. The annotations overlap and the two final annotations would be: ‘Brain Region: VB’ and ‘Neuron: VB Neurons’. The second example shows a more complex example of the annotation process. Several overlapping Brain Regions have been annotated as well as two mentions of neuron types and one mention of species (referred to as model organism for greater clarity elsewhere in this paper).

An active learning criterion can help to identify which sequences are informative in an unlabelled dataset. These informative sequences are useful for training a high performance named entity recognition model. In our annotation process, we used an uncertainty based active learning criterion: ‘normalized entropy’ (Kim et al. 2006). The uncertainty based methods have been shown to lead to an early increase in performance when annotating selected sequences from a dataset (Settles and Craven 2008). This criterion assumes that the most informative unlabelled sequences are those for which the current named entity recognizer is most uncertain about the predicted sequence of labels. Given an unlabelled sequence ***x*** and a set *Y* of labels, ***y*** is defined as a label sequence for ***x*** and *y*_*i*_ ∈ *Y* is the corresponding named entity label of *x*_*i*_. *x*_*i*_ is the *i*-th word in sequence ***x***. *P*(***y***| ***x***) is the conditional probability of ***y*** under the sequence ***x***. The steps that measure the informativeness of ***x*** are as follows. In the first place, we use the current model to predict the label sequence for ***x*** and select the top-*N* best predicted label sequences {***y***^(**1**)^, ***y***^(**2**)^, …, ***y***^(***N***)^} with the highest probabilities {***P***(***y***(^(**1**)^| ***x***), ***P***(***y***^(**2**)^| ***x***), …, ***P***(***y***^(***N***)^| ***x***)}. Secondly, the entropy of sequence ***x*** is defined based on these conditional probabilities of the top-*N* label sequences as shown in Eq. 1.

1 $$ \mathrm{Entropy}\left(\boldsymbol{x}\right)=-\sum \limits_{i=1}^N\frac{P\left({\boldsymbol{y}}^{\left(\boldsymbol{i}\right)}|\boldsymbol{x}\right)}{\sum \limits_{j=1}^NP\left({\boldsymbol{y}}^{\left(\boldsymbol{j}\right)}|\boldsymbol{x}\right)}{\mathit{\log}}_2\left[\frac{P\left({\boldsymbol{y}}^{\left(\boldsymbol{i}\right)}|\boldsymbol{x}\right)}{\sum \limits_{j=1}^NP\left({\boldsymbol{y}}^{\left(\boldsymbol{j}\right)}|\boldsymbol{x}\right)}\right] $$

Thirdly, we use the formula from Eq. 2 to normalize the entropy to [0,1].

2 $$ \mathrm{Normalized}\_\mathrm{Entropy}\left(\boldsymbol{x}\right)=\frac{\mathrm{Entropy}\left(\boldsymbol{x}\right)}{-{\mathit{\log}}_2\frac{1}{N}} $$

An unlabelled sequence with a high normalized entropy score is considered informative and its corresponding annotated label sequence is likely to be useful to improve the performance of the current model. In our experiments, we set *N* = 3 and use CRF++ to obtain the top-*N* predicted label sequences with conditional probabilities for each unlabelled sequence in a dataset.

### Rules and Dictionaries

We implemented a number of dictionary and rule based methods of identifying the entities that we are interested in.

We used the online resource InterLex^4^ and extracted the names and synonyms of all entities belonging to the following classes: Brain Region (http://uri.interlex.org/base/ilx_0109835), Neuron Type (http://uri.interlex.org/base/ilx_0107497), Model Organism (http://uri.interlex.org/base/ilx_0107049), Ion Channel (http://uri.interlex.org/base/ilx_0105699), Ion Current (http://uri.interlex.org/base/ilx_0111888) and Ion Conductance (http://uri.interlex.org/base/ilx_0102463). The list of ion conductances was smaller than the corresponding list of ion currents, so we adapted our list of ion currents to represent names of ion conductances augmenting our list of ion conductances (using the replacement ‘current’ to ‘conductance’, where appropriate). We also converted acronyms for currents by replacing ‘I’ with ‘g’ (e.g., ‘IH’ to ‘gH’). We used the NERSuite Dictionary matcher to match entities to their dictionary entries.

We used regular expressions to identify Neuron types, Ion Channels, Ion Conductances and Ion Currents. These were developed based on the typical names of an ion current. Before we applied the regular expression, we first employed a syntactic parser to identify the noun phrases in a text. Each noun phrase was evaluated to determine whether it contained a matched regular expression. If so, we assigned the whole noun phrase to be of the matched annotation type. The regular expressions were as follows:

- Neuron types:. *(neuron(e?s)?)|(cells?)(. *)
- Ion Current:. *(current)s?
- Ion Channel:. *(channel)s?
- Ion Conductance:. *(conductance)s?

To detect scientific Units, we employed a gazetteer which provided information about each unit in question. The gazetteer we employed contained 24 unit names (volt, gram, etc.), which could be combined with 19 standard prefixes (milli, kilo, etc.) to give complex unit names (milligram, kilometre, etc.). Short forms of units and prefixes were also included to give abbreviated unit names (mg, km, mm, etc.). UTF-8 encoding was used in our tools to handle special characters when they appeared in units.

Experimental Values were detected by first identifying any potential values in a text using the regular expression:

$$ \backslash \mathrm{d}+\left(,\backslash \mathrm{d}\left\{3\right\}\right)\ast \left(\backslash .\backslash \mathrm{d}+\right)? $$

The regular expression is made up of three distinct parts that can be used to detect numbers. The first part ‘\d+’ detects an integer value made up of at least one decimal number. ‘1’, ‘23’, or ‘345’ would all be matched by this part of the regular expression. The second part of the regular expression ‘(,\d{3})*’ detects subsequent groups of thousands. There may be 0, 1, or more of these. This works in tandem with the first part of the regular expression to detect numbers such as ‘1100’, ‘23,400,500’ or ‘345,600,700,800’. Note that improperly formatted thousands separators will not be recognised (e.g., ‘1,23’, ‘34,567,1’). The final part of the regular expression ‘(\.\d+)?’ allows for the detection of decimal numbers. This may be part of the overall number, or it may be absent. It works in tandem with either the first part of the regular expression, or both the first and second part to detect numbers such as ‘1.1’, ‘1100.543’. N.b., that improperly formatted decimals or hierarchical numbers will not be recognised as numbers (e.g., ‘1.1.1.1’ would not be recognised as a number).

Any numbers that were detected with this regular expression were linked to scientific units that had been discovered using the gazetteer approach. A number was linked to a value if it was within the same noun phrase as that value. This accounted for longer dependencies, such as ranges or lists of values where the unit is only present at the end. E.g., “10-20mv”, “15, 30 and 45 millimetres”. Negative numbers were detected in post-processing to avoid confusing them with numbers occurring as parts of lists.

In addition to the above we used Acromine (Okazaki and Ananiadou 2006; Okazaki et al. 2010) to link common acronyms to their expanded forms. Acromine draws upon the whole of MEDLINE to generate acronyms for common terms. If any of the expanded forms were annotated elsewhere in the text, we assigned the same annotation type to the acronym in question.

### Conditional Random Field

CRF is a statistical machine learning technique, which is designed to transform a sequence of input labels into a sequence of output labels. The CRF takes the context of the sequence into account during classification. This is different to a classical classification algorithm (such as linear regression, or support vector machine), which considers each instance independently. For example, if we were classifying segments of audio data as speech phonemes, then we might learn that certain phonemes occur more frequently together than others. The CRF is capable of learning these associations automatically. The user only needs to provide training data and a set of appropriate features to allow the algorithm to identify and learn these associations.

We used the NERSuite implementation of the conditional random field for named entity recognition. NERSuite relies on several features for each token in the data. An example of the features that were created by NERSuite is shown in Table 2, where the sentence ‘*Abstract: Whole-cell voltage recordings were made in vivo in the ventral posterior medial nucleus (VPM)*’ has been annotated with features by NERSuite.

**Table 2:** The features that were created by NERSuite at the token level

Begin	End	Word	Lemma	POS	Chunk	Dictionary
0	8	Abstract	Abstract	NN	B-NP	O
9	10	:	:	:	O	O
11	16	Whole	Whole	JJ	B-NP	O
17	22	-cell	-cell	JJ	I-NP	O
23	30	voltage	voltage	NN	I-NP	O
31	41	recordings	recording	NNS	I-NP	O
42	46	were	be	VBD	B-VP	O
47	51	made	make	VBN	I-VP	O
52	54	in	in	FW	B-ADVP	O
55	59	vivo	vivo	FW	I-ADVP	O
60	62	in	in	IN	B-PP	O
63	66	the	the	DT	B-NP	O
67	74	ventral	ventral	JJ	I-NP	B-BrainRegion
75	84	posterior	posterior	JJ	I-NP	I-BrainRegion
85	91	medial	medial	JJ	I-NP	I-BrainRegion
92	99	nucleus	nucleus	NN	I-NP	I-BrainRegion
100	101	(	(	(	O	O
102	105	VPM	VPM	NN	B-NP	B-BrainRegion
106	107	)	)	)	O	O

The features in Table 2 are as follows:

- Begin: The starting offset of each word in terms of its character index.
- End: The index of the character immediately after the word. This ensures that subtracting the value for begin from the value for end will give the length of the token.
- Word: The raw wordform as it appeared in the text
- Lemma: The base form of the word – found by dictionary lookup (e.g., ‘were’ becomes ‘be’, ‘made’ becomes ‘make’).
- POS: the part of speech of the word provided by the GENIA tagger (Tsuruoka and Tsujii. 2005). This indicates whether a noun, verb, adjective, adverb or something else was present. The codes used above are as follows: NN=Noun, NNS = Plural noun, VBD = Past tense of verb, VBN = Past participle of verb, DT = Determiner, JJ = adjective, IN = Preposition and FW = Foreign word.
- Chunk: the syntactic chunk that the text currently represents. B- I- and O represent Begin, Inside and Outside, respectively. NP = Noun Phrase, VP = Verb Phrase, ADVP = Adverb Phrase, PP = Prepositional Phrase.
- Dictionary: We added dictionary features for all entities apart from the Value entity (where it was not possible to create a valid dictionary). We used the dictionaries that we described in the section above. For Units, we used the names of the units derived from the gazetteer. B, I and O are as above. In the example, we have shown the dictionary entries for brain regions, hence B-BrainRegion indicates the beginning of a brain region mention and I-BrainRegion indicates that the token is still inside the Brain Region mention. When annotating other entity types, the appropriate dictionary is used.

Finally, the data was split into train, test and validation sets (although the validation set was only used for parameter tuning in the Deep Learning NER). For training and evaluation purposes, we added a further column of data to represent the gold standard annotation that our annotators had judged to be the correct set of labels for the entity type that was being classified. We trained a separate CRF model for each entity type and then tested each model on the held out test data.

### Deep Learning NER

Long short-term memory (LSTM) is a variant of the recurrent neural network (RNN), which uses a neural network to encode contextual information about a sequence. To capture features describing both past (left context) and future (right context) information associated with a given sequence, forward and backward LSTM (bi-directional LSTM) has been applied to many different classification tasks (Miwa and Bansal 2016; Habibi et al. 2017; Dligach et al. 2017) and performs very well compared with approaches relying on hand-crafted features. In our work, we use a model based on the work of Lample et al. (2016). We re-implemented their first model using Chainer (Tokui et al. 2015).

To represent the output of our system, we needed a scheme that allowed us to state which tokens corresponded to an annotation. One annotation may cover multiple tokens. For example, “ventral posterior medial nucleus” is one brain region, but it covers four tokens. We chose to use the BIO scheme, which encodes information for each token labelling it as ‘B’ – the beginning of an annotation, ‘I’ – inside an annotation, or ‘O’ – outside an annotation. Every annotation starts with ‘B’, the subsequent tokens that are part of that annotation are labelled as ‘I’ and any tokens before or after the annotation that are not part of it are labelled as ‘O’. This means that the neural network only has to learn to place each token into one of these three categories, simplifying the task.

**Fig. 2:**
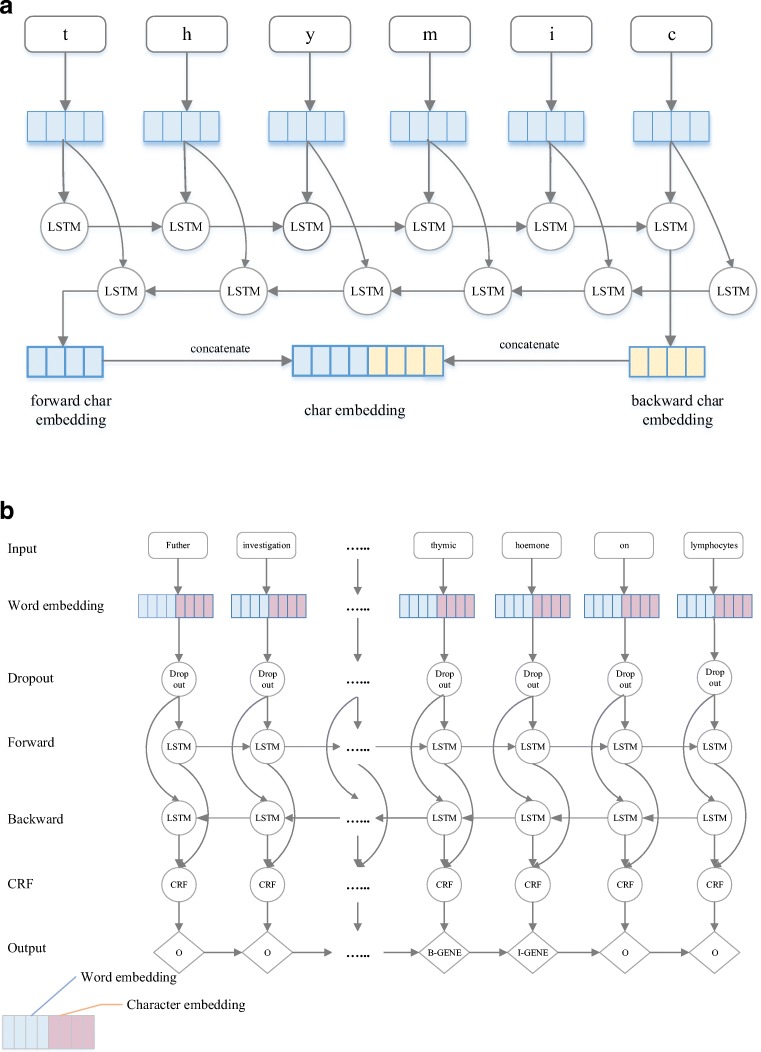
**a** The neural network architecture that we used to produce the character embeddings. The input to the neural network is an embedding for each character. The neural network operates on each character embedding using two LSTM units (one capturing context before the token, and one capturing context after the token). The final output (after all characters have been processed for the word) of the two LSTM units is concatenated to give a final character embedding for the word. This type of embedding captures orthographic information. The embedding is fed into the neural network architecture shown in Fig. 2b. **b** The neural network architecture that we used to produce our output sequence of BIO labels. The input layer consists of the word embedding for each token, concatenated with the character embedding that is generated as described in Fig. 2a. The concatenated word-character embedding is passed through a dropout layer, which regularises the embedding by selectively dropping connections in the network. This is then passed through a forward-backward LSTM layer, which captures contextual information from the sequence before and after the given token. The output of both layers (forward and backward) is concatenated and passed to a CRF, which is trained to give an output corresponding to the correct label in BIO format

Our model consists of four layers: embedding layer, character bidirectional LSTM layer, word bidirectional LSTM layer and CRF layer, as shown in Fig. 2b. Taking sequences and pre-trained word embeddings as the input, the model outputs sequences labelled with tags. Details are described next.

1. Embedding layer

Given a sequence s = {w_1_, w_2_, w_3_, ….., w_n_}, w_i_ represents a word in this sequence. Firstly, a unique ID is assigned to each word in the training data set to represent itself. This unique ID allows the computer to efficiently store each word as a value. Similarly, all the characters are mapped to a dictionary and each character in this dictionary is described by a unique integer. Therefore, each word can be represented by a word ID and a series of character IDs. Subsequently, two linear functions are separately applied to each word and its characters to generate two outputs: word vectors and a character-based word matrix. The row dimension of the character-based word matrix equals the length in characters of its word. Contrary to a randomly initialised character-based word matrix, pre-trained word embeddings (Chiu et al. 2016) which were trained from a 2.7 billion word corpus from PubMed is applied to initialize each word vector. More specifically, words included in the pre-trained embeddings are assigned with corresponding vectors in our word embeddings while unknown words (those that do not appear in the embedding) are randomly initialized using a linear function. The pre-trained embeddings are trained with the skip-gram model on a large number of unannotated abstracts from PubMed. We chose to use biomedical specific embeddings as these have been shown to be more effective than general purpose domain embeddings for some tasks (Chiu et al. 2016).

2) Character bidirectional LSTM

Previous work has demonstrated orthographical features contribute to named entity recognition (Limsopatham and Collier 2016; Bhasuran et al. 2016). Given a word, instead of using hand-crafted affix features, we use the Bidirectional LSTM (BiLSTM) to acquire character representations for each word. Suppose the character matrix of a given word is Cnxm where n and m mean the length of this word and the dimension of the character vector, respectively. We firstly feed this matrix to the forward LSTM and use the hidden state of the last character to represent left context information of the word. In the same way, the backward LSTM is employed to each column of the matrix in reverse order to generate right context information of this word. Next, we concatenate these two vectors as the character representation of this word. This is depicted in Fig. 2a.

3) Word bidirectional LSTM

For each word in the given sequence, the character representation acquired from the previous layer, concatenated with the word representation, constitute the input to this layer. To avoid over-fitting, we apply a common regularisation technique called “dropout” for each word before sending to BiLSTM. Then, forward and backward context representation acquired from the BiLSTM layer is connected, acting as the input to the CRF layer.

4) CRF layer

Context representation is fed into the CRF layer, which considers neighbour information to decode the best label sequence.

The hyperparameters used in our model are shown in Table 3. We used grid search to train the learning and dropout rate on the validation set. All other parameters were set using the default values specified by Chainer.

**Table 3:** The hyper parameters that were used during training of our model

Hyper Parameter	Value
Epochs	20
Batch Size	10
Character embedding dimension	25
Word embedding dimension	200
Dropout rate	0.5
Character-based word embedding	250
Optimization method	Adam
Learning Rate	0.013
Weight decay	0.0001
Pre-trained word embeddings	PubMed-shuffle-win-2.bin (Chiu et al. 2016)

## Results

In this section, we present the results of the corpus creation process and the evaluation we performed on the corpus using the named entity recognition tools that we developed.

### Corpus Creation

Inter annotator agreement was assessed using the F1 metric. This metric allows us to see how likely the two annotators were to agree on a given annotation class. It is more sensitive to disagreements than using percentage of agreement, as the F1 score is calculated as the harmonic mean of precision and recall and therefore will be low if either of these metrics is low. In addition, F1 score only takes into account instances where annotators have agreed on a named entity, or where they have disagreed. It does not reward annotators for agreeing on words in a text which are not marked as named entities. This is important as named entities are sparse in a text and rewarding agreement on the absence of a named entity would overly bias the results. We present the results of the corpus creation process in Table 4.

**Table 4:** Final statistics on our corpus. We show a level of agreement above 0.8 for all but two categories. Agreement is reported as F1 score

Entity	Agreement	Number Double Annotated	Total
Brain Region	0.891	480	1055
Neuron Type	0.825	309	767
Model Organism	0.846	150	299
Ion Channel	0.639	83	201
Ion Current	0.904	137	339
Ion Conductance	0.810	50	76
Value	0.784	211	594
Unit	0.902	151	507
All	0.837	1571	3838

In Table 4, we have also included the number of double annotated entities, as well as the total number of annotated entities. One annotator annotated 500 sentences from abstracts and 500 further sentences from full papers. The other annotator annotated 200 sentences from the abstracts and 200 sentences from the full papers to allow us to calculate agreement.

Some examples of annotations from the full corpus are included below:

1. “Gramicidin -perforated patch-clamp recordings were made from slices of the *suprachiasmatic nucleus* (*SCN*) of adult *rats*”
*(Brain Region: “suprachiasmatic nucleus”, “SCN”. Model Organism: “rats”)*

(2). The emergence of depolarizing GABA(A)-mediated responses in a subset of *SCN neurons* at night can be ascribed to a depolarizing shift in GABA(A) reversal potential.
*(Neuron Type: “SCN neurons”)*

(3). Whole-cell *I(Ts)* amplitude was increased when Ba2+ was substituted for Ca2+ as the charge carrier.
*(Ion Current: “I(Ts)”)*

(4). FRB in this model is favored by enhancing *persistent g(Na)* and also by measures that reduce [Ca(2+)](i) or that reduce *the conductance of g(K(C))*
*(Ion Conductance: “persistent g(Na)”, “conductance of g(K(C))”)*

(5). Using in situ hybridization, we have localized central and peripheral nervous system expression of three transcripts (*alpha1G*, *alpha1H*, and *alpha1I*) of the *T-type calcium channel* family (*CaVT*).
*(Ion Channel: “alpha1G”, “alpha1H”, “alpha1I”, “T-type calcium channel”, “CaVT”).*

(6). The effect of bicuculline (*12.5 microM*) on the spontaneous firing rate of *SCN neurons* during the night was heterogeneous due to the mixture of depolarizing and hyperpolarizing GABA(A)-mediated inputs during this period.

*(Units: “microM”, Experimental Value “12.5 microM”, Neuron Type: “SCN neurons”, Brain Region “SCN”).*

### Named Entity Recognition

Once we had developed the corpus, we were able to use it to train and test tools for named entity recognition. We tested 3 methods as outlined earlier in this paper, representing a variety of techniques in the area of named entity recognition. In Table 5, we have highlighted the best performing system in terms of Precision, Recall and F1 score in bold font.

**Table 5:** The results of our methods to identify the entities in our corpus. We see that Deep Learning is the strongest approach for all entities except for Value

Entity	Rules and Dictionaries			Conditional Random Field			Deep Learning NER		
	P	R	F1	P	R	F1	P	R	F1
Brain Region	0.324	0.304	0.314	**0.880**	0.772	0.822	0.856	**0.833**	**0.844**
Neuron Type	0.225	0.336	0.269	0.864	0.673	0.757	**0.878**	**0.760**	**0.814**
Model Organism	0.599	0.365	0.435	**0.927**	0.776	0.844	0.860	**0.878**	**0.869**
Ion Channel	0.322	0.244	0.278	0.643	0.563	0.600	**0.737**	**0.875**	**0.800**
Ion Current	0.128	0.109	0.118	0.853	0.580	0.690	**0.872**	**0.680**	**0.764**
Ion Conductance	0.056	0.092	0.070	**1.000**	0.222	0.364	0.929	**0.722**	**0.813**
Value	0.264	0.320	0.289	**0.897**	**0.839**	**0.867**	0.895	0.828	0.860
Unit	0.256	0.544	0.348	**0.915**	0.942	0.929	0.904	**0.957**	**0.930**
Average	0.272	0.289	0.265	**0.872**	0.671	0.734	0.866	**0.817**	**0.837**

To produce the results in Table 5, we used the rules and dictionaries to generate named entities for the entire corpus, as these did not require any training data. We then split the full corpus into three partitions ‘train’, ‘test’ and ‘validate’ in the ratio 70%:15%:15%. We trained the CRF and Deep learning NER for each entity type using the training set, validated the hyper-parameters for the Deep Learning NER on the validation set and then tested both the CRF and Deep Learning NER on the test set. We performed no further tuning after we had generated the results on our test set. We used the same train and test set for both the CRF and Deep Learning NER, as this gives a fair comparison of their relative performance.

It is clear to see from Table 5 that the dictionary and rule based methods did not perform well, only capturing a fraction of cases. This implies that the dictionaries and rules we used were not able to capture the variety of named entities in the full corpus. The conditional random field performs much better than the rules and dictionaries, indicating that contextual information is important for the recognition of the entities that we are interested in. The deep learning NER performs better again than the CRF in most instances. The deep learning NER attains the highest F1 score for all entity classes, apart from the ‘Experimental Value’ class. The CRF approach often attains higher precision than the deep learning NER (Brain Region, Model Organism, Ion conductance, Value, Unit). However, this is outweighed by improved recall when using the deep learning NER to give higher F1 scores overall.

In the neuroinformatics literature, there are only two approaches that are directly comparable to ours. Firstly, French et al. (2009) developed a large corpus of brain region mentions and built a custom CRF to identify their named entities. French et al. used context features which determined whether a given word was likely to occur before or after an annotation, as well as features encoding structural information about the tokens. This work was improved upon by Richardet et al. (2015a), where features encoding the presence of species and measurements were added to the CRF model, leading to a slight increase in performance as shown in Table 6. Our Deep Learning NER, described in this paper, differs from these algorithms as it uses a different classification framework. Whereas the two previous approaches used hand crafted features, we use features that are statistically learnt from the text through deep learning. To compare our best approach to this previous work, we trained the Deep Learning NER on the corpus provided by French et al. (2009). We show the results reported by the previous work, alongside our results in Table 6.

**Table 6:** Our results compared to previously published NER tools for Brain Regions. To allow us to compare our results on a level playing field with the prior work, we have presented two different evaluation protocols (as these were both presented in the previous work). Strict matching indicates that an annotated named entity is only considered to be correct if it directly matches the gold standard data, i.e., both boundaries must be exactly the same. Relaxed matching indicates that an annotated named entity is only considered to be correct if the boundaries overlap the boundaries of an entity in the gold standard data. The boundaries of the annotated entity may or may not directly coincide with the boundaries of the gold standard entity

Approach	Strict			Relaxed		
	P	R	F1	P	R	F1
French et al. (2009)	0.813	0.761	0.786	0.916	0.857	0.886
Richardet et al. (2015a)	0.846	0.788	0.816	0.884	0.810	0.846
Deep Learning NER	0.821	0.815	0.818	0.934	0.927	0.931

### Hardware Specification

The following machines were used in the course of this research. For the deep learning experiments, we used a server with 2 Six-core Intel Sandybridge CPUs, 32GB RAM and two Tesla K20c, 5GB GPU memory, 2496 CUDA cores, CUDA compute capability 3.5. For the CRF and NER experiments we used a Macbook Pro with a 2.5 GHz Intel Core i7, 16GB RAM. We did not make use of the graphics card for these experiments. For the active learning experiments, we used a single core 2.60 GHz server with 5GB of RAM.

## Discussion

Our corpus is small, containing annotated versions of 1000 sentences. Typically, tool performance is directly correlated to corpus size, and so the larger the corpus the better. However, we have taken the approach of using active learning in our work. Active learning allows us to extract only the most relevant parts of the documents that we are interested in. So, in our full corpus, we have 15 fully annotated abstracts (the seed set), the most relevant 500 sentences from 160 further abstracts and the most relevant 500 sentences from 15 full papers. The hypothesis of active learning is that by using selection criteria to extract the most useful sentences, we can gain a similar performance to manually annotating the full data set but with reduced effort. The strong performance of our data-driven approaches implies that our active learning paid off, and that we were able to create a useful set of tools with a small corpus. If we were to perform further annotation to augment the corpus size (either using active learning or not), we would expect to see the performance of our tools increase. The limitation on performing further annotation is the time and cost associated with attaining high quality annotations from experts working in the field, as we have done in this work.

The agreement between the annotators is generally very high as shown in Table 4, attaining a mean agreement of 0.837. This shows that the annotators agreed in most of the cases. It also indicates that the annotators broadly agreed about which entities should and should not be annotated (i.e., that true positives far outweighed false positives and false negatives). The agreement dips slightly for ion channel, indicating that they found this class more difficult to agree upon than other classes. In the cases where disagreements arose between the annotators, we first attempted to resolve the dispute by discussing the discrepancies with the annotators. Where disagreements persisted, we deferred to the first, more senior, annotator’s judgements.

The corpus we have created is principally useful for the training and testing of named entity recognition tools in the neuroscience domain. The corpus may be used by others to train their own tools, as well as to evaluate the performance of new tools with the same purpose. Other researchers may also wish to extend the corpus with new entity or relation types or indeed more documents to further improve the performance of the trained NER tools. To maximise reuse, the corpus, including train-test splits, is available via the supplementary work. In addition to this, the tools we have described are available via the OpenMinTeD platform as components developed for the Argo Text Mining workbench (Rak et al. 2012).

It is clear from results in Table 5 that the rules and dictionaries approach did not perform as well as could have been expected. There are two factors that may have influenced their performance. Firstly, the resource that we adopted to generate our dictionaries (Neurolex) clearly did not contain sufficient cases to accurately capture the wide scope of named entities that were present in our corpus. This is unsurprising as the premise of our work is that manual curation of entities from the neuroscience literature is an arduous task, which requires many person hours and can be prone to mistakes. This shows the need for automation for the curation of entities in the neuroscience literature. The second factor that is likely to have influenced the results of the rules and dictionaries approach is the wide variation in the form of each term. Different authors use different ways of referring to the same entities and may use differing abbreviations for a term. These variant forms are difficult to capture with a dictionary, especially as they were not recorded and standardised in the resource that we used to get our lists of entities. The regular expressions also seemed to offer little in the way of performance. The expressions we used may have been overly simple and more complicated regular expressions or e.g., context-sensitive grammars, could be used to capture other forms in which entities appeared.

We employed two machine learning approaches for named entity recognition, namely NERSuite CRF and a deep learning system, which also used a CRF as its output layer. It is clear from our results in Table 5 that the two machine learning approaches outperformed the resource driven approach based on dictionaries and regular expressions. The difference between these two approaches is that the NERSuite CRF and deep learning NER are both capable of learning features from the data that are useful for classifying the entities that we are interested in. For the NERSuite CRF, we start with a fixed set of features which are defined as part of the NERSuite system. The CRF is then capable of learning from the pre-labelled training data, which we provided to give the associations between the features and the labelled data. Typically, the CRF is very good at learning contextual features, which makes it very suitable for cases of named entity recognition where the terms typically occur with high variability of form, but low variability of context, as is the case for our entity types in neuroscience. For example, an author will typically mention the same types of words and word patterns in the context of neuron types or brain regions and these act as clues that can be used to aid the CRF in classification. The NERSuite CRF performed well for all named entities apart from ion conductance, where a high recall but low precision was observed. This is likely due to the low number of ion conductances (*n* = 76) in our data.

The deep learning NER system again led to an increase in performance over the NERSuite CRF. This is in line with findings throughout the natural language processing literature, where deep learning methods have been shown to outperform traditional methods. The deep learning method takes the contextual associations that can be discovered by the learning algorithm a step further. Instead of relying on a set of pre-defined features (which may or may not be relevant to the specific NER case), the deep learning method instead uses word embeddings (which represent the context of a word as a dense information vector). These embeddings, along with character embeddings which encode orthographic relations, are passed through a bi-directional LSTM, which extracts and combines the relevant parts of the information vector to create a new information vector that contains the most relevant parts of the information from the original embeddings. This process means that complex relations can be learnt between words in a sentence based not only on the words themselves, but also the words that may occur within the context of those words found in the sentence. The dense information vector is passed to a CRF layer as with the NERSuite CRF, which is capable of learning structured relations between the words. The increased performance of the Deep Learning NER over the NERSuite CRF indicates that the Deep Learning NER was able to access more information about each named entity, and use this information to make better judgments on which words corresponded to named entities. This is expected as the Deep Learning NER uses word embeddings as its data source, which encode deep contextual information about each word and are richer than the features passed to the NERSuite CRF. The Deep Learning NER outperforms the NERSuite CRF in terms of recall for seven out of eight classes (all except for ‘Experimental Value’). However, for precision, the Deep Learning NER only outperforms the NERSuite CRF in 3 out of eight cases (see Table 5). When these two statistics are combined to give F1 score, the Deep Learning NER outperforms the NERSuite CRF in all cases, except for Experimental Value. This indicates that a small drop in precision for some classes is outweighed by a much larger gain in recall. A drop in precision indicates that the false negative rate has increased, i.e., that the classifier is missing some elements that should be classified as the given type. An increase in recall indicates that the false positive rate has decreased, i.e., that the classifier is better at identifying things that should not be classified as the given type.

To highlight the differences between the machine learning methods and the dictionary-based method we have collected several examples from our annotated dataset that highlight the differences between a rule-based approach and our best performing data-driven approach (the Deep Learning NER). The sentences focus on the annotation of brain regions and are presented in Table 7.

**Table 7:** A comparison of dictionary based recognition of brain regions to the Deep Learning NER system. The manual annotation of the texts, which we used to judge our system’s performance against, is also included

Text	Dictionary	Deep Learning NER	Manual Annotation
1. curve partially around the rostral pole of the ventral posteromedial nucleus (VPM)	“ventral posteromedial nucleus”,“VPM”	“rostral pole of the ventral posteromedial nucleus”, “VPM”	“rostral pole of the ventral posteromedial nucleus”, “VPM”
2. and the other population projected mainly to orbital or cingulate areas.	No brain regions detected.	“orbital or cingulate areas”	“orbital or cingulate areas”
3. from the rat ventrobasal complex (VB) and posterior nucleus (POm).	“ventrobasal complex”, “nucleus”	“ventrobasal complex”, “VB”, “posterior nucleus”, “POm”	“ventrobasal complex”, “VB”, “posterior nucleus”, “POm”

Table 7 shows three examples of sentences that were annotated as part of our evaluation by both the dictionary based system and the Deep Learning NER. In each case, the Deep Learning NER system has been able to get the same results as our gold standard manual annotations (even though it has never seen these examples before), whereas the dictionary-based system has made some mistake. In the first example, the Deep Learning NER system and the human annotator both extracted the text “rostral pole of the ventral posteromedial nucleus” as the brain region that was being mentioned. However, the dictionary-based system missed the first part of the annotation, only getting ‘ventral posteromedial nucleus’. Whilst this is usually a brain region, in this case it is not the brain region being described and would be misleading if it were accepted as correct. The dictionary based system was not able to detect this complex term, instead deferring to a simpler term which was already in the dictionary. The Deep Learning NER system has been able to use information about the context and structure of the sentence to correctly assign the whole part of the annotation as a brain region. In the second example, the human annotator and the Deep Learning NER system both picked out the phrase ‘orbital or cingulate areas’ as a brain region of interest. The dictionary-based system, however, did not find any brain region in this area. In this case, the specific phrase is not in the dictionary we used, and therefore was not picked up by the dictionary-based system. In the final example, the human annotator and the Deep Learning NER system have both picked out the following brain regions: “ventrobasal complex”, “VB”, “posterior nucleus” and “POm”. The dictionary-based system, however, has picked out the following brain regions: “ventrobasal complex” and “nucleus”. Whilst the first brain region picked by the dictionary-based system is correct, it has missed both the acronyms and has only found half of the second annotation (missing the word “posterior”). We were able to detect some acronyms using the dictionary-based system (as seen in example 1), however this did not have as high accuracy as for the Deep Learning NER. For sentence 3, the acronyms were not part of the dictionary and could not be resolved to the text, therefore they were not annotated. The Deep Learning NER system has been able to learn something about the context of these acronyms that has allowed it to correctly identify them as brain regions.

In this work, we have not addressed the problem of linking entities to an ontology. A curator must take an entity that has been identified and either link it to an existing node within the ontology, or create a new node and associations therewith. Whilst it is relatively simple to link existing entities to a knowledge base through dictionary matching, it is historically difficult to automatically incorporate data from text mining algorithms into an ontology (Spasic et al. 2005). It would be interesting to further extend this work to look at automatically incorporating the results of the Deep Learning NER that we have proposed into an existing knowledge base.

## Conclusion

We have developed a new corpus containing named entities for neuroscience. Our work represents the broadest attempt to perform statistical named entity recognition for neuroscience to date (in terms of the total number of named entity types that we have explored). We used state of the art named entity recognition tools in the form of our Deep Learning NER system to develop text mining tools to detect the named entities that we have described in this paper. Further to this, we have compared our work with the existing NERs, where we were able to show that our method compared favourably with previous techniques according to both a strict and a relaxed matching protocol. We have shown that by following our techniques, a researcher can create competitive NER tools within a specific domain using a very small set of annotations. This is in contrast to previous approaches, which have required large scale annotation efforts to get comparable performances. To extend this work, we will look to incorporate further entity types (including synapses and modelling parameters) in our annotation scheme, provide normalization to terms from NIFSTD, and look at identifying relations between the entities to further aid curation. Finally, we will integrate the output of these tools with NeuroCurator to semi-automate the annotation process and allow faster curation of larger corpora to support ongoing and future large-scale brain modelling projects, meta-analyses, and data-driven neuroscience in general.

## Information Sharing Statement

The corpus and the NER tools are available via GitHub (See: https://github.com/nactem/TM4NS). In addition, we have incorporated our tools into the OpenMinTeD platform: https://services.openminted.eu/landingPage/application/3eb79c9d-ab73-41fc-823a-ecaa688c7f1b

## Electronic supplementary material

ESM 1 (PDF 252 kb)

## Footnotes

1
https://taku910.github.io/crfpp/

2 http://nersuite.nlplab.org/

3
https://uima.apache.org/ruta.html

4
https://neuinfo.org/interlex/dashboard
