# Supplementary material for: A Text Mining Pipeline Using Active and Deep Learning Aimed at Curating Information in Computational Neuroscience
Source: Neuroinformatics. 2018 Nov 15;17(3):391–406. doi: 10.1007/s12021-018-9404-y (PMC6594987; doi:10.1007/s12021-018-9404-y)
Supplement: Supplementary file 1 — (PDF 252 kb) [file 12021_2018_9404_MOESM1_ESM.pdf]

OMTD Neuroscience Annotation Guidelines

Table of contents

**Table of contents**	**1**
**Generalities**	**1**
**Brain Region**	**4**
**Ionic Current**	**4**
**Ion Channel**	**5**
**Ionic Conductance**	**5**
**Neuron**	**6**
**Model Organism / Species**	**6**
**Scientific Values**	**7**
**Scientific Units**	**8**

Generalities

**Directives - acronyms**
Acronyms should be annotated separately to the text that is being referred to. I.e. in the text below, TRN would be a separate annotation to 'thalamic reticular nucleus'.
**Examples (annotating brain regions)**
The inhibitory sources in the **thalamic nuclei** are local interneurons and neurons of the **thalamic reticular nucleus (TRN)**. Within the **TRN**, the neurons are [...]

**Directives - specific entities vs generic class**
Only the reference to a specific member of a class (e.g., neurons) should be annotated. Not the general case. E.g., in the example below, 'cells' in red should not be annotated.
**Examples (annotating neurons)**
The inhibitory sources in the thalamic nuclei are **local interneurons** and **neurons of the thalamic reticular nucleus (TRN)**. These two classes of **cells** are [...]

**Directives - adjective forms and hyphens**
The adjective form of an entity should not be annotated.<br><br>In a related idea, entity names that are used as qualifier using an hyphen should not be annotated.
**Examples (annotating brain regions)**
The cortical interneurons are [...].<br>The interneurons of the **cortex** are [...].<br>[...] gave rise to intra-nRt collaterals [...]<br><br>**Examples (annotating ionic currents)**

[...] **T-type Ca2+ current**, and the T-current-dependent low threshold Ca2+ spike [...]

[...] the T-type Ca(2+) channel-dependent high frequency burst firing of action potentials displayed by [...]

Examples

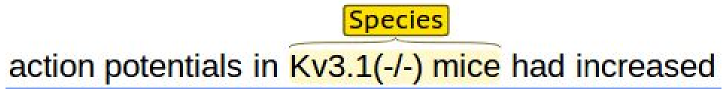

[Note: Kv3.1 is not annotated as a channel since it is part of the single “word” Kv3.1(-/-)]

Directives - entity imbrication

When an entity is a specific case of a more inclusive entity within an imbricated structure, both the specific entity and the inclusive entity should be annotated.

Examples (annotating brain regions, delimiting the annotations with [ ] to show the imbricated structures)

The **[ventroposteral lateral nucleus of the [thalamus]]** [...].

Directives - lists

Lists of entities should be treated as separate entities.

Examples

sensory ganglia, pituitary, and dentate gyrus granule neurons
Neurons: (1) sensory ganglia granule neurons
(2) pituitary granule neurons
(3) dentate gyrus granule neurons

Directives - split annotations

Annotations should be split in segments when necessary. Generally this applies to lists

Examples

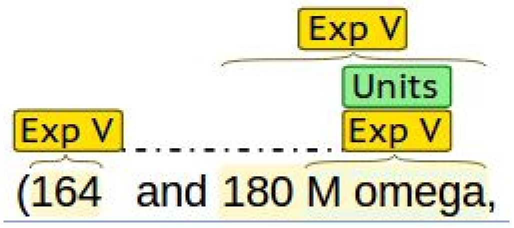

Brain Region

**Definition**
Any phrase describing an area of the brain. This includes for example a cortical layer. This includes area mentioned by their function (e.g., the **somatosensory area**) but exclude the mention of the system (e.g., the somatotosensory system) or of a “representation” (e.g., the shoulder representation).
**Examples**
Low-threshold Ca2+ spikes (LTS) are an indispensable signaling mechanism for neurons in areas including the **cortex**, **cerebellum**, **basal ganglia**, and **thalamus**.<br><br>The inhibitory sources in the **thalamic nuclei** are local interneurons and neurons of the **thalamic reticular nucleus**.

Ionic Current

**Definition**
An ion current is the influx and/or efflux of ions through an ion channel. (Source: Wikipedia) We do not annotate excitatory/inhibitory postsynaptic currents as ionic currents (e.g. “NMDAR current”).
**Examples**
A **hyperpolarization-activated cation conductance** contributes to the membrane properties of a variety of cell types.

The steady-state conductances of depolarizing **Ih** (**hyperpolarization-activated cationic current**), **IT** (**low-threshold calcium current**), and **INaP** (**persistent sodium current**) move the membrane potential away from the reversal potential of the leak conductances.

Ion Channel

Definition

An ion channel is a transmembrane molecule allowing under certain conditions the exchange of some ions between the intra and the extracellular environment. These can be referred to either by the name of their molecule or by the name of the gene coding this molecule. Reference to only sub-domain of an ion channel should not be annotated (i.e., only reference to the channel as whole are annotated), unless they refer share a clear relationship with a type of ion channel. For example alpha1G (a type of alpha subunit) is used as synonym of Cav3.1 ion channels (http://channelpedia.epfl.ch/ionchannels/85). In general, alpha sub-units can give their name to the channel type, not beta sub-units (at least for soduim and calcium). In case of genetically modified animals referred to as gene_name-/- where the gene_name is associated with a knock-down ion channel, we annotate gene_name as (a reference to an) an ion channel (see examples).

Examples

Unexpectedly, however, we found that both WT and KO mice for **CaV3.1**, the gene for **T-type Ca2+ channels** in TC neurons [...].

Both tonic and burst spikes were observed in **CaV3.1+/+** TC cells, whereas only tonic spikes were detected in **CaV3.1−/−** TC cells.

Ionic Conductance

Definition

The conductance associated with the electrical model of an ion channel or of a ionic current. We also use this entity to annotate ionic resistances, since these two concepts are directly related (resistance = 1/conductance). When annotating conductances, we do not annotate gmin or gmax because these do not refer to the conductance itself but to the minimal or maximal value of this conductance.

Examples

1 Conductance values obtained by dividing the current by the driving force were plotted against voltage for each cell and fitted to a single Boltzmann function of the form

Io_Cdt

2 gKir(V)=gmin+(gmax−gmin)/{1+exp[(V−V1/2)/k]},

3 (1)

Io_Cdt Ionic_Conductance

4 where gKir(V) is the conductance of the barium-sensitive component at a given voltage value; gmax and gmin are the maximum and minimum conductances, respectively; V is the voltage value at every point during the ramp protocol; V1/2 is the half-activation voltage; and k is the slope factor.

Neuron

Definition

An electrically excitable cell that processes and transmits information through electrical and chemical signals. (Source: Wikipedia)
Neurons are polarized cells with defined regions consisting of the cell body, an axon, and dendrites, although some types of *neurons* lack axons or dendrites. (Source: NeuroLex)
When the neuron is names as "neuron type X of region Y" the part "of region Y" should be included in the neuron name.

Examples

Our data demonstrate that key somatodendritic electrical conduction properties are highly conserved between **glutamatergic thalamocortical neurons** and **GABAergic thalamic reticular nucleus neurons** and that these properties are critical for LTS generation.

The inhibitory sources in the thalamic nuclei are **local interneurons** and **neurons of the thalamic reticular nucleus**.

Model Organism / Species

Definition

A term referring to a species' name. These are typically used in experiments and may be referred to via an informal name as well as the formal latin name. We use the term “species” in its more generic case and, accordingly, it should also be used to annotate more specific entities like strains. Similar, it should also be used to annotate class of species (e.g., rodent). Note that some annotations concerns the species in a somewhat implicit manner. This is the case when talking about some strain and its wild-type counterpart; wild-type is not in itself a species but is referring to species entity (i.e., the wild-type counterpart of an experimental strain previously mentioned). For the same reason, “normal rat” will be annotated (including the “normal” qualificative) when it is use to contrast to another strain (e.g., GAERS) and is implicitly used as a synonym for “wild type”.

Examples

We found that in both **Wistar rats** and **GAERS**, the proportion of interneurons was significantly higher in the LGN than in the VPM and VPL.

Unexpectedly, however, we found that both **WT** and **KO mice** for CaV3.1, the gene for T-type Ca2+ channels in TC neurons, exhibit typical waxing-and-waning sleep spindle waves at a similar occurrence and with similar amplitudes and episode durations during non-rapid eye movement sleep.

Action potential trains in **Kv3.1(-/-)** were associated [...]

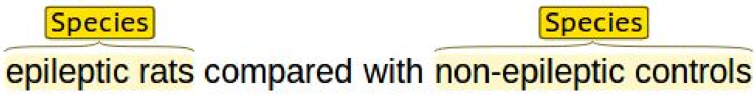

Scientific Values

Definition

A quantifiable number (including the unit, if any are present), denoting a value. This may occur as a range (e.g., -100 to -40 mV). It might also happen in case of list of measurements all related to the same entities (i.e., they are repeated measurements). However, two values separated by an “and” referring to two different entities (see the e.g. with “0.13 and 0.20 ms respectively” below, referring to measurements in two different cell types) should be annotated as separated values. Sample size should not be annotated. In text where the ~ symbol is used as a synonym as “approximately” (e.g., ~20 mm), the ~ symbol should be included in the annotation.

Examples

In the VPM, the proportion of interneurons was **4.2%** in Wistar and **14.9%** in GAERS; in the VPL the values were **3.7%** for Wistar and **11.1%** for the GAERS.

Whereas the time course of Na+ channel activation (**-30 to +40 mV**) was similar, the deactivation kinetics (**-100 to -40 mV**) were faster in BCs than in PCs.

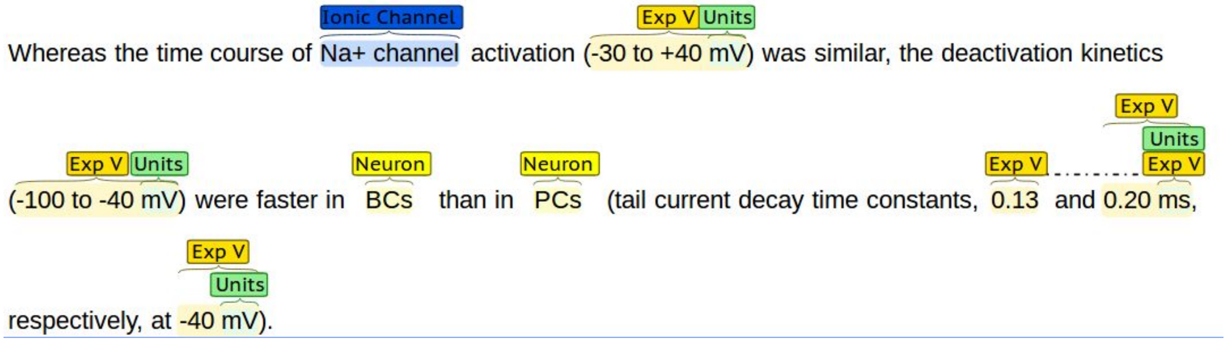

Scientific Units

Definition

Units which describe a scientific quantity, possibly as defined by the SI. These will often occur as abbreviations. Of note, a unit can in some case be a noun that is not typically an entity, as in “20 **neurons**”.

Examples

In the VPM, the proportion of interneurons was 4.2**%** in Wistar and 14.9**%** in GAERS; in the VPL the values were 3.7**%** for Wistar and 11.1**%** for the GAERS.

Whereas the time course of Na+ channel activation (-30 to +40 **mV**) was similar, the deactivation kinetics (-100 to -40 **mV**) were faster in BCs than in PCs (tail current decay time constants, 0.13 and 0.20 **ms**, respectively, at -40 **mV**).
